# IMPDH2 promotes colorectal cancer progression through activation of the PI3K/AKT/mTOR and PI3K/AKT/FOXO1 signaling pathways

**DOI:** 10.1186/s13046-018-0980-3

**Published:** 2018-12-05

**Authors:** Shiyu Duan, Wenqing Huang, Xiaoting Liu, Xuming Liu, Nana Chen, Qiong Xu, Yukun Hu, Wen Song, Jun Zhou

**Affiliations:** 1 Department of Pathology, Nanfang Hospital, Southern Medical University, 1838 Guangzhou North Road, Guangzhou, 510515 China; 2 Department of Pathology, School of Basic Medical Sciences, Southern Medical University, 1838 Guangzhou North Road, Guangzhou, 510515 China; 3 Guangdong Provincial Key Laboratory of Molecular Oncologic Pathology, Southern Medical University, 1838 Guangzhou North Road, Guangzhou, 510515 China; 4 School of Pharmaceutical Sciences, Guangdong Provincial Key Laboratory of New Drug Screening, Southern Medical University, Guangzhou, 510515 China

**Keywords:** *IMPDH2*, Colorectal cancer, *Proliferation*, Cell cycle, *EMT*

## Abstract

**Background:**

Inosine 5′-monophosphate dehydrogenase type II (IMPDH2) was originally identified as an oncogene in several human cancers. However, the clinical significance and biological role of IMPDH2 remain poorly understood in colorectal cancer (CRC).

**Methods:**

Quantitative real-time polymerase chain reaction (qPCR), western blotting analysis, the Cancer Genome Atlas (TCGA) data mining and immunohistochemistry were employed to examine IMPDH2 expression in CRC cell lines and tissues. A series of in-vivo and in-vitro assays were performed to demonstrate the function of IMPDH2 and its possible mechanisms in CRC.

**Results:**

IMPDH2 was upregulated in CRC cells and tissues at both mRNA and protein level. High IMPDH2 expression was closely associated with T stage, lymph node state, distant metastasis, lymphovascular invasion and clinical stage, and significantly correlated with poor survival of CRC patients. Further study revealed that overexpression of IMPDH2 significantly promoted the proliferation, invasion, migration and epithelial-mesenchymal transition (EMT) of CRC cells in vitro and accelerated xenograft tumour growth in nude mice. On the contrary, knockdown of IMPDH2 achieved the opposite effect. Gene set enrichment analysis (GSEA) showed that the gene set related to cell cycle was linked to upregulation of IMPDH2 expression. Our study verified that overexpressing IMPDH2 could promote G1/S phase cell cycle transition through activation of PI3K/AKT/mTOR and PI3K/AKT/FOXO1 pathways and facilitate cell invasion, migration and EMT by regulating PI3K/AKT/mTOR pathway.

**Conclusions:**

These results suggest that IMPDH2 plays an important role in the development and progression of human CRC and may serve as a novel prognostic biomarker and therapeutic target for CRC.

## Background

Colorectal cancer (CRC) is one of the most common types of malignancies worldwide [1], and its incidence and mortality rates are continuously increasing. Despite the fact that improvements have been made in diagnostic methods and therapeutic strategies, the overall prognosis of CRC patients still remains pessimistic. Hence, it is desperately needed to improve our identification of the molecular mechanisms underlying CRC progression and to develop more efficient therapeutic methods of managing CRC.

Inosine5′-monophosphate dehydrogenase (IMPDH) is a rate-limiting enzyme which catalyzes the nicotinamide adenine dinucleotide (NAD^+^)-dependent oxidation of inosine monophosphate (IMP) to xanthosine monophosphate (XMP), which is an essential step in de novo biosynthesis of guanine nucleotides [2]. IMPDH is a key regulator of the intracelluar guanine nucleotide pool, demonstrating its importance for DNA and RNA synthesis. Human IMPDH is a tetramer composed of approximately 55 kDa monomers [3] and has two distinct isoforms, IMPDH1 and IMPDH2, with an 84% similarity in their amino acid sequence [4]. IMPDH1 is generally expressed in normal human leukocytes and lymphocytes, whereas IMPDH2 is generally upregulated in tumor tissues and proliferating cells [5–7]. Most importantly, the increase in total IMPDH activity is mainly attributed to increased expression of IMPDH2 [4]. Nowadays, isoforms of IMPDH, particularly IMPDH2, have been of particular interest to oncologists due to its roles in regulation of cell proliferation, cell differentiation, and chemoresistance [4, 8–11].

Accumulating evidence reveals that IMPDH2 was significantly elevated in multiple types of tumor cells and associated with cancer progression and poor prognosis of tumor patients [12–14]. For instance, increased IMPDH2 expression was observed in human melanoma cell lines [15], human ovarian tumors [13], human leukemic cell lines [7] and multiple myeloma cells [16]. A study by Fellenberg et al. showed that IMPDH2 could be served as a promising candidate for the stratification of osteosarcoma patients into low- and high-risk groups [8]. Furthermore, inhibition of IMPDH2 activity increased sensitivity to methotrexate in HT29 human colon cancer cells [17], and induced growth arrest of human multiple myeloma cells [16]. To date, however, the biological role of IMPDH2 in CRC progression and its molecular mechanisms have not been well elucidated.

In our study, IMPDH2 was shown to be highly expressed in CRC cell lines and tissues. A series of in vitro and in vivo assays revealed that overexpressing IMPDH2 dramatically promoted the proliferation, invasion and migration, tumorigenicity and epithelial–mesenchymal transition (EMT) of CRC cells, while knockdown of IMPDH2 had the opposite effect. We further demonstrated that IMPDH2 overexpression accelerated G1/S phase cell cycle transition by inducing increased expression of cyclin D1 and Ki-67 and downregulation of p21Cip1 and p27Kip1. More importantly, G1/S phase cell cycle transition was triggered by IMPDH2 through activation of AKT activity, downregulation of mTOR and FOXO1 transcriptional activity. Additionally, inhibition of the mTOR pathway could induce suppression of invasion, migration and EMT in IMPDH2-overexpressed cells. These findings suggest that IMPDH2 plays a potential oncogenic role in CRC progression and represents a promising prognostic marker of this disease.

## Methods

### Cell culture

Human embryonic kidney 293 T cells, normal human colon epithelial cells (FHC (CRL-1831)) and seven human CRC cell lines, including HCT116, SW620, M5, SW480, HT29, DLD-1 and LoVo were obtained from a cell bank at the Chinese Academy of Sciences (Shanghai, China). All cells were authenticated by short tandem repeat (STR) profiling after receipt and were propagated for less than 6 months after resuscitation. All CRC cell lines were cultured in RPMI 1640 medium (Gibco, Gaithersburg, MD, USA) with 10% fetal bovine serum (HyClone, Logan, USA) and 100 U/ml penicillin/ streptomycin (Gibco). These cell lines were maintained in a humidified chamber containing 5% CO_2_ at 37 °C.

### Tissue preparation

For western blotting and quantitative real-time PCR (qPCR) analyses, 34 pairs of fresh CRC tissues and matched adjacent normal colorectal tissues from primary CRC patients were obtained in operation from Nanfang Hospital, Southern Medical University (Guangzhou, China). Paraffin-embedded specimens of 214 primary CRC patients who undergone elective operation were collected from Nanfang Hospital between February 2009 and June 2011. None of these patients received any preoperative chemotherapy or radiotherapy. The stage of disease was determined according to the tumor size, lymph node involvement and distant metastasis (pTNM) classification system [18]. Complete follow-up, ranging from 1 to 96 months, was available for the cohort of 214 patients, and the median survival was 53 months. The study was approved by the Ethics Committee of Nanfang Hospital, Southern Medical University and all aspects of the study comply with the Declaration of Helsinki. Written informed consent was obtained from all patients.

### Immunohistochemistry

The expression level of IMPDH2 protein in 214 pairs of paraffin-embedded CRC tissues and matched adjacent normal colorectal tissues was examined by immunohistochemistry (IHC). The sections were heated, deparaffinized, rehydrated and placed insodium citrate buffer (pH = 6.0) for antigen retrieval. Then the slides were immersed in 3% hydrogen peroxide to inhibit the endogenous peroxidase activity. After rinsing three times, the sections were incubated with primary antibody (rabbit anti-IMPDH2, 1:800 dilution, #ab131158; Abcam, Cambridge, UK) overnight at 4 °C, followed by treatment with secondary antibody (anti-rabbit IgG, 1:2000 dilution, #7074; Cell Signaling, Danvers, MA, USA) for 40 min at 37 °C. After being stained with 3,3-diaminobenzidine (DAB), the slides were counterstained with Mayer’s haematoxylin, dehydrated and mounted.

IHC scoring based on the staining intensity and the proportion of positive tumor cells was performed by two independent pathologists blinded to the clinical data. The staining intensity was scored as 0 (negative), 1 (weak), 2 (medium), 3 (strong). The extent of staining was scored as 0 (0%), 1 (1–25%), 2 (26–50%), 3 (51–75%) and 4 (76–100%), according to the percentage of the positive staining areas in relation to the whole tumor area or the entire section for the normal sample. The sum of the intensity and extent scores was used as the final staining score (0–7) for IMPDH2. For statistical analysis, a final staining score of ≥3 was considered to be high, and the scores of < 3 as low expression of IMPDH2.

### RNA extraction and qPCR

Total RNA from cultured cells and fresh tissues was extracted with Trizol regent (Invitrogen, Calsbad, CA). Synthesis of cDNA was performed by using the PrimeScript RT reagent Kit (Promega, Madison, WI, USA). The SYBR Premix EX Taq™ (Takala, Dalian, China) was used for quantitative real-time PCR (qPCR) operated with an ABI 7500 Real-Time PCR system (Applied Biosystems, Foster City, USA). The primer sequences used to amplify IMPDH2 were: 5′- GTTTCTGCGGTATCCCAATC -3′ (forward) and 5′- CGAGCAAGTCCAGCCTAT-3′ (reverse). GAPDH was used as an endogenous control. Relative gene expression was determined by the comparative 2-ΔΔCT method.

### Western blotting analysis

Proteins from cell and tissue lysates were separated by SDS-polyacrylamide gel electrophoresis (PAGE) and electrotransferred onto a polyvinylidene difluoride (PVDF) membrane (Pall Corp, Port Washington, NY). Then the membranes were blocked with 5% skimmed milk and incubated using primary antibodies against IMPDH2 (1:1000 dilution), anti-GAPDH, anti-GSK3β, anti-p-GSK3β, anti-AKT, anti-p-AKT (Ser473), anti-FOXO1, anti-p-FOXO1, anti-mTOR, anti-p-mTOR (Cell signaling Technology, Beverly, MA), E-cadherin (1:1000 dilution,#SAB4503751; Sigma Aldrich), β-catenin (1:1000 dilution, #C2206; Sigma Aldrich), Vimentin (1:1000 dilution, #V6630; Sigma Aldrich), Snail (1:1000 dilution, #SAB1306281; Sigma Aldrich), followed by incubation with the appropriate secondary antibodies (anti-rabbit IgG, 1:3000 dilution, #7074; Cell Signaling). An enhanced chemiluminescence (Pierce, Rockford, IL, USA) was used to detect signals.

### Lentiviral transduction and transfection

Overexpression and short hairpin RNA (shRNA) -induced downregulation of IMPDH2 in CRC cells were achieved using the GV367 and GV248 shRNA lentiviral vector (genechem, Shanghai, China). The human shRNA sequences to inhibit IMPDH2 expression are listed as follows: IMPDH2 shRNA: GGACAGACCTGAAGAAGAA (genechem, Shanghai, China). The vectors were packaged in 293 cells. Recombinant lentiviruses were produced by transient transfection of HEK293T cells. Then, transduced cells were selected for 7 days with 0.6 mg/mL puromycin. Protein and mRNA of transfer cells were taken for qPCR and western blotting analyses.

### Cell proliferation assay

1 × 10^3^ cells were seeded on 96-well plates and cultured for 24 h.2-(2-Methoxy-4-nitrophenyl)-3-(4-nitrophenyl)-5-(2,4-disulfothenyl)-2H-tetrazolium salt (CCK-8, Dojindo, Rockville, USA) solution was added to each well and incubated for 2 h, and then the absorbance of each well was measured at 450 nm with a Microplate Autoreader (Bio-Rad, Hercules, CA, USA). The experiment was performed with three replicates.

### Colony formation assay

Cells were plated on 6-well plates (200 cells/well) and maintained for 2 weeks. The colonies were stained with 1% crystal violet for 30 s after fixation with 4% paraformaldehyde for 30 min. The number of colonies, defined as > 50 cells/colony, was counted. Three independent experiments were performed.

### Cell wound healing assay

Cell motility was measured with wound healing assay. 1.2 × 10^6^ Cells were seeded on 6-well tissue culture plates and incubated for 24 h. Scratch wounds were produced by a 10 μL pipette tube, after three washes with cold PBS. And the spread of wound closure was observed after 0 and 48 h, singly. Images were taken to assess the level of migration in each group of transfected cells. Cell motility was quantified by measuring the distance between the advancing margins of cells in three randomly selected microscopic fields (× 200) at each time point.

### Transwell migration assay

2 × 10^5^ cells suspended in serum-free media were placed in the upper compartment of 8-μm-pore Transwells (BD Biosciences, San José, CA, USA) and 10% FBS as a chemo-attractant was filled in the lower compartment. The cells were incubated at 37 °C for 2 days. The successfully translocated cells were stained with 0.5% crystal violet for 15 min and calculated in five randomly chosen fields (× 200) under a microscope.

### Immunofluorescent assay

Cells were seeded on Confocal plates per well for 48 h and incubated with primary antibodies against E-cadherin (1:200 dilution, #ab1416; Abcam, Cambridge, UK) and Fibronectin (1:200 dilution, #ab2413; Abcam, Cambridge, UK), then followed by incubation with green goat anti rabbit and red goat anti rabbit antibodies. After counterstaining with 4′,6-diamidino-2-phenylindole (DAPI; Sigma), an Olympus FV1000 confocal laser-scanning microscope (Olympus America Inc., NY, USA) was used to take images.

### Animal experiments

Balb/C-nu/nu athymic nude mice (3–4 weeks old) were obtained from the Laboratory Animal Centre of Southern Medical University, which is certified by the Guangdong Provincial Bureau of Science. To explore the function of the IMPDH2 gene in colorectal tumour growth in vivo, 2 × 10^6^ cells suspended of LoVo with stable overexpression of IMPDH2 and HCT116 with stable knockdown of IMPDH2, or mock cells, were respectively injected into left and right bilateral hind leg subcutaneous of mice. All mice were housed and maintained under specific pathogen-free conditions, and all experiment were approved by the Use Committee for Animal Care and performed in accordance with institutional guidelines. These mices were allocated randomly into each of the four groups (*n* = 6 per group). Tumour volume was estimated from two perpendicular axes using a digital calipers every two days (volume = (length×width^2^)/2 and tumour were also photographed. The primary tumour was removed surgically, fixed, paraffin-embedded, and sectioned. The sections were observed under a microscope after haematoxylin and eosin (H&E) staining.

For the in vivo metastasis assay, 2 × 10^5^ of HCT116 cells stably expressing control or IMPDH2 shRNA in a volume of 150 μl in the PBS were injected into nude mice (n = 6 per group) via the tail vein, respectively. Lung metastases of tumor cells were observed 2 months post-injection. The lungs were removed by dissection away from adjacent organs and fixed with 10% formalin. The consecutive tissue sections were obtained and stained with haematoxylin-eosin (H&E) to observe the metastatic nodules of lungs under a microscope. All animal experiments were conducted in strict accordance with the principles and procedures approved by the Committee on the Ethics of Animal Experiments of Southern Medical University.

### Gene set enrichment analysis (GSEA)

To gain insight into IMPDH2-mediated molecular pathways in colorectal cancer, GSEA was performed using the Broad Institute GSEA version 4.0 software. The Cancer Genome Atlas (TCGA) database consisted of 644 colorectal cancer tissues samples was downloaded from the TCGA (TCGA, https://cancergenome.nih.gov/). The gene sets used for the enrichment analysis were downloaded from the Molecular Signatures Database (MsigDB, http://software.broadinstitute.org/gsea/index.jsp). The gene sets with a false discovery rate (FDR) less than 0.25 were considered as significantly enriched.

### Statistical analysis

All statistical analyses were carried out using the SPSS 19.0 statistical software package. In at least three independent experiments, the data were presented in terms of the mean ± SD. The Student t-test and the one-way ANOVA test were carried out for qPCR. Comparisons between groups for statistical significance were carried out with a 2-tailed paired Student t-test. The correlation between the expression of IMPDH2 and clinicopathologic factors was evaluated using Pearsonʼs chi-squared (χ2) test. For patients with different levels of IMPDH2 expression, the survival curves were plotted using the Kaplan-Meier method and compared using the log-rank test. The Cox proportional hazard model was used for multivariate analysis. *P*<0.05 was considered statistically significant.

## Results

### IMPDH2 is up-regulated in CRC

**Fig. 1:**
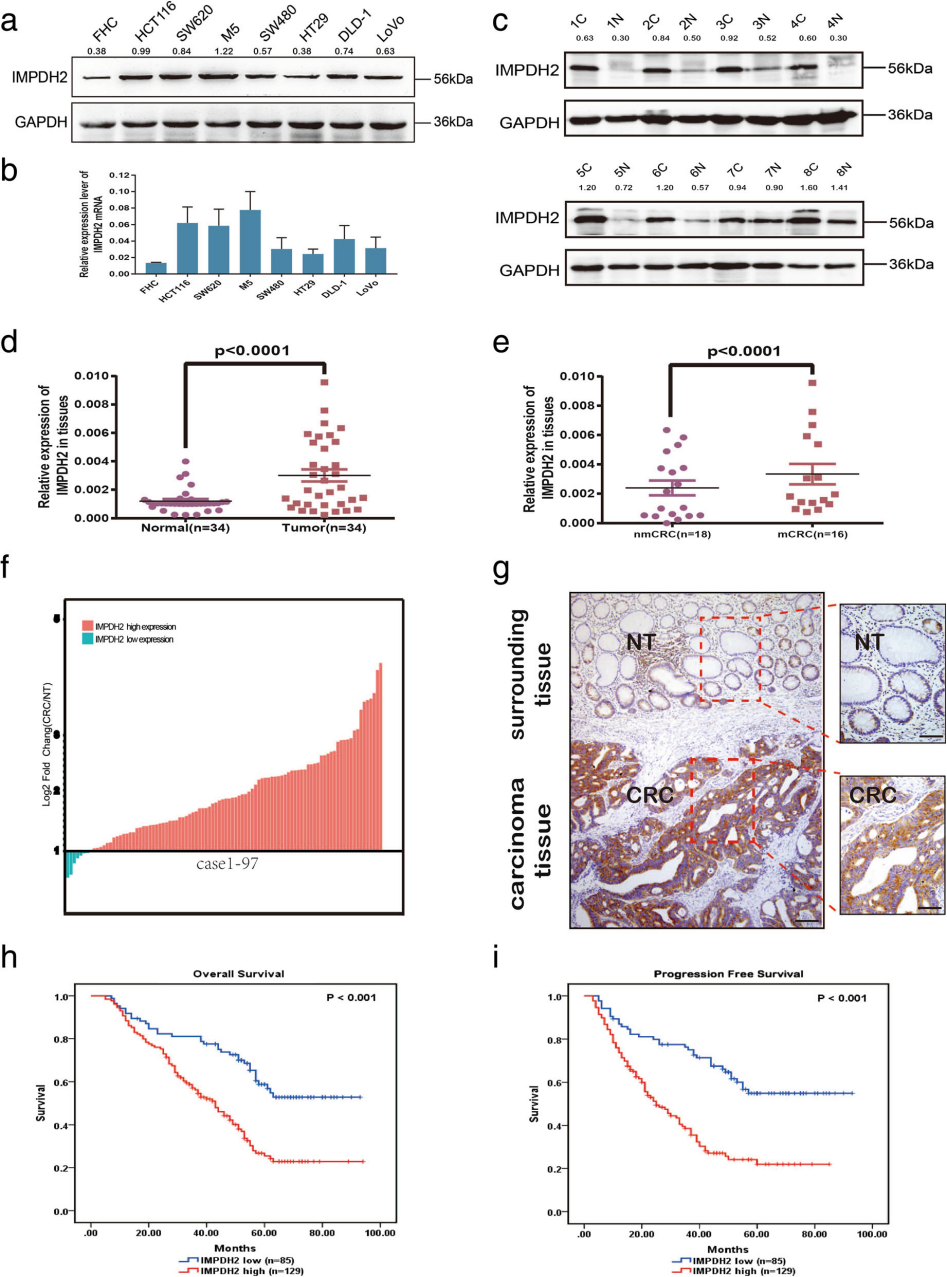
The relationship between IMPDH2 expression and poor prognosis of CRC. (**a** and **b**) The relative expression of IMPDH2 protein and mRNA in normal human colon epithelial cells (FHC) and seven CRC cell lines (HCT116, SW620, M5, SW480, HT29, DLD-1 and LoVo) by western blotting and qPCR. Mean ± SD (*n* = 3). **(c)** The expression of IMPDH2 protein in 8 surgical CRC tissues and their paired adjacent normal tissues by western blotting. **(d)** The expression of IMPDH2 mRNA in 34 pairs of fresh CRC tissues and matched adjacent normal tissues using qPCR. **(e)** The IMPDH2 expression in CRC tissues with or without metastases. nmCRC denotes CRC tissues without metastases (*n* = 18); mCRC denotes CRC tissues with lymph node metastases (*n* = 16). **(f)** The expression of IMPDH2 mRNA in 97 paired human CRC tissues and their adjacent normal mucosa tissues from TCGA dataset. IMPDH2 expression was normalized to GAPDH and expressed relative to the match adjacent normal tissues. **(g)** Representative images of IMPDH2 expression in CRC tissues and paired adjacent normal tissues (NT) by immunohistochemistry, Scale bars, 200 μm and 50 μm, respectively. **(h and i)** Kaplan-Meier survival analysis of the association between IMPDH2 expression and overall survival or progression-free survival in 214 CRC patients

Real-time quantitative PCR (qPCR) and western blotting analysis showed that the levels of IMPDH2 expression were varied in FHC cells and seven CRC cell lines including HCT116, SW620, M5, SW480, HT29, DLD-1 and LoVo. IMPDH2 expression was the higher in M5, HCT116 and SW620 cells but the lower in FHC, HT29, SW480 and LoVo cells (Fig. 1a and b).

In order to further determine the expression level of IMPDH2 in clinical samples of human CRC, we performed data mining in publicly available CRC datasets using the TCGA database. Upregulation of IMPDH2 mRNA was found in 90 of all 97 CRC samples when compared to the control samples (Fig. 1f). To verify this observation, qPCR was employed to measure the expression level of IMPDH2 mRNA in 34 fresh CRC tissues and paired adjacent normal tissues. As described in Fig. 1d, the expression of IMPDH2 mRNA in CRC tissues was significantly higher than that in matched adjacent non-malignant tissues (*P* < 0.05). Interestingly, compared with those who had no lymph node metastasis, patients with lymph node metastasis had higher mRNA level of IMPDH2 in CRC tissues (*P* < 0.05, Fig. 1e). Furthermore, western blotting showed a higher expression level of IMPDH2 protein in 8 pairs of CRC tissues than in the corresponding normal colorectal tissues (Fig. 1c). In addition, immunohistochemistry (IHC) was performed to examine the expression of IMPDH2 protein in 214 paraffin-embedded CRC tissues. We found that IMPDH2 protein was mainly located in the cytoplasm of benign and malignant epithelial cells (Fig. 1g), and 129/214 (60.28%) of CRC tissues exhibited a significantly higher level of IMPDH2 compared with the matched normal tissues (Table 1). Collectively, these data suggests that IMPDH2 is remarkably elevated in CRC.

**Table 1:** Relationship between IMPDH2 expression level and clinical pathological parameters of CRC

IMPDH2 expression					
Characteristics	*n* = 214	Low (%)	High (%)	*P* value	x^2^
Gender				0.083	3.014
Male	136	60(44.1)	76(55.9)		
Female	78	25(32.1)	53(67.9)		
Age (years)				0.154	2.033
<55	106	37(34.9)	69(65.1)		
≥ 55	108	48(44.4)	60(55.6)		
Tumor site				0.626	0.936
Proximal colon	47	17(36.2)	30(63.8)		
Distal colon	37	13(35.1)	24(64.9)		
Rectum	130	55(42.3)	75(57.7)		
Tumor size (cm)				0.253	1.305
<5	116	42(36.2)	74(63.8)		
≥ 5	98	43(43.9)	55(56.1)		
Tumor differentiation				0.758	0.554
Well	81	34(42.0)	47(58.0)		
Moderate	101	40(39.6)	61(60.4)		
Poor	32	11(34.4)	21(65.6)		
T stage				0.048	6.057
T1–2	59	31(52.5)	28(47.5)		
T3	140	50(35.7)	90(64.3)		
T4	15	4(26.7)	11(73.3)		
Lymph node state				< 0.001	13.525
Positive	88	22(25.0)	66(75.0)		
Negative	126	63(50.0)	63(50.0)		
Distant metastasis				0.026	4.962
Positive	38	9(23.7)	29(76.3)		
Negative	176	76(43.2)	100(56.8)		
Lymphovascular invasion				0.018	5.551
Positive	73	21(28.8)	52(71.2)		
Negative	141	64(45.4)	77(54.6)		
Clinical stage				0.001	15.697
1	53	29(54.7)	24(45.3)		
2	63	31(49.2)	32(50.8)		
3	60	16(26.7)	44(73.3)		
4	38	9(23.7)	29(76.3)		

### High IMPDH2 expression is associated with several aggressive features and poor prognosis of CRC

To explore whether IMPDH2 expression is associated with the clinicopathological characters of CRC, the clinical data from these 214 CRC patients were analyzed. As summarized in Table 1, high expression of IMPDH2 protein was positively associated with T stage (*P* = 0.048), lymph node state (*P* < 0.001), distant metastasis (*P* = 0.026), lymphovascular invasion (*P* = 0.018) and clinical stage (*P* = 0.001) in CRC patients. However, there was no significant correlation between IMPDH2 expression and other clinicopathological parameters (*P* > 0.05, Table 1).

Furthermore, Kaplan-Meier survival analysis showed that patients with high IMPDH2 expression had shorter overall survival and progression-free survival than those exhibiting low IMPDH2 expression (*P* < 0.001, Fig. 1h and i). In addition, Cox regression analyses revealed that lymph node state, distant metastasis and IMPDH2 expression might be recognized as independent prognostic factors for CRC patients (Table 2).

**Table 2:** Univariate and multivariate Cox regression analysis of prognostic factors in 214 CRC patients for overall survival

Variable	Univariate analysis				Multivariate analysis		
	HR	95% CI	*P*-value		HR	95% CI	*P*-value
Overall survival							
Gender	1.257	0.878–1.800	0.211				
Age (years)	0.900	0.632–1.282	0.559				
Tumor site	0.963	0.777–1.193	0.730				
Tumor size(cm)	0.792	0.553–1.136	0.205				
Tumor differentiation	1.187	0.915–1.539	0.197				
Lymph node state	2.673	1.867–3.826	< 0.001		1.728	1.166–2.561	0.006
Distant metastasis	6.534	4.285–9.961	< 0.001		4.993	3.198–7.796	< 0.001
IMPDH2 expression	2.427	1.633–3.607	< 0.001		1.891	1.248–2.866	0.003

### Overexpression of IMPDH2 promotes the proliferation, invasion, migration and tumourigenesis of CRC cells

**Fig. 2:**
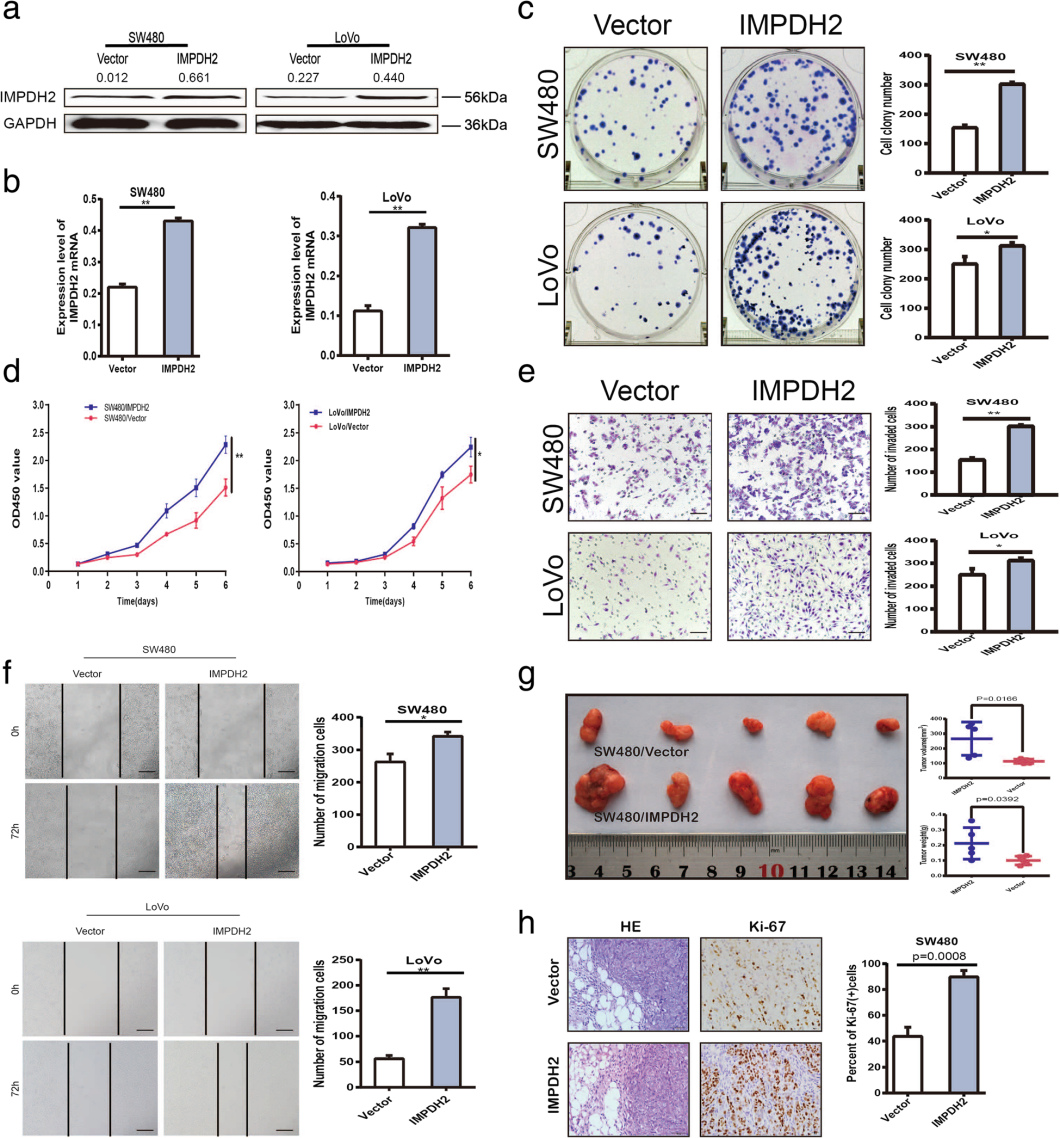
Overexpression of IMPDH2 promotes proliferation, migration and invasion of CRC cells and accelerates tumour growth in the nude mouse model. (a and b) Overexpression of IMPDH2 was confirmed at the protein and mRNA level in SW480 and LoVo cells by western blotting and qPCR. Mean ± SD (n = 3). **(c and d)** IMPDH2 overexpression promoted proliferation ability of SW480 and LoVo cells as determined by colony formation and CCK8 assays. Mean ± SD (n = 3). **(e)** IMPDH2 overexpression significantly promoted the invasion ability of SW480 and LoVo cells by the transwell assay. Representative photographs (left) and quantification (right) are shown. The number of cells that invaded through the extracellular matrix after 24 h was counted in five randomly selected microscopic fields. Mean ± SD (*n* = 3). Scale bars, 100 μm. **(f)** IMPDH2 overexpression significantly promoted the migration ability of SW480 and LoVo cells by cell wound healing assay. Images were taken at 0 h, 24 h, 48 h and 72 h. The number of migrated cells was counted (right). Mean ± SD (*n* = 3). Scale bars, 200 μm. **(g)** IMPDH2 overexpression promoted tumour growth in the nude mouse model by xenograft growth assay. Gross observation of xenograft tumour size (left). Plot of tumour volume and weight over time (right). (**h**) H&E and Ki-67 staining of a xenograft tumour. The percent of Ki-67 positive cells was shown (right). Each error bar represents the mean ± SD of three replicate samples. Scale bars, 50 μm and 20 μm. *P < 0.05; **P < 0.01

In order to investigate the possible functional roles of IMPDH2 in CRC progression, two stable IMPDH2-overexpressed CRC cell lines, SW480/IMPDH2 and LoVo/IMPDH2 were established. SW480 and LoVo transduced with empty lentiviral vectors were used as negative controls. Western blotting and qPCR analysis confirmed a significant increase of IMPDH2 expression in SW480/IMPDH2 and LoVo/IMPDH2 cells compared with the expression level of IMPDH2 in control cells (Fig. 2a and b). The colony formation and CCK8 assays showed that overexpressing IMPDH2 promoted the proliferation of SW480 and LoVo cells (Fig. 2c and d). Moreover, overexpression of IMPDH2 remarkably enhanced the invasive and migratory abilities of SW480/IMPDH2 and LoVo/IMPDH2 cells, detected by the transwell and wound healing assays (*p* < 0.05, Fig. 2e and f).

Additionally, to elucidate the effect of IMPDH2 on tumor growth in vivo, xenograft growth assays were performed in nude mice by subcutaneous injection of LoVo/IMPDH2 cells and control cells. As shown in Fig. 2g, the xenograft tumours were detected on the ninth day after injection. Tumors from the LoVo/IMPDH2 group grew significantly faster than those from the control group at day 42 (*P* < 0.05, Fig. 2g). Compared to the tumors formed by the control cells, the tumors derived from IMPDH2-overexpressed CRC cells exhibited a higher cell proliferation index as shown by Ki-67 staining (Fig. 2h), demonstrating that IMPDH2 plays a crucial role in the growth of CRC cells in vivo.

### Downregulation of IMPDH2 suppressed the proliferation, invasion, migration and tumorigenicity of CRC cells

**Fig. 3:**
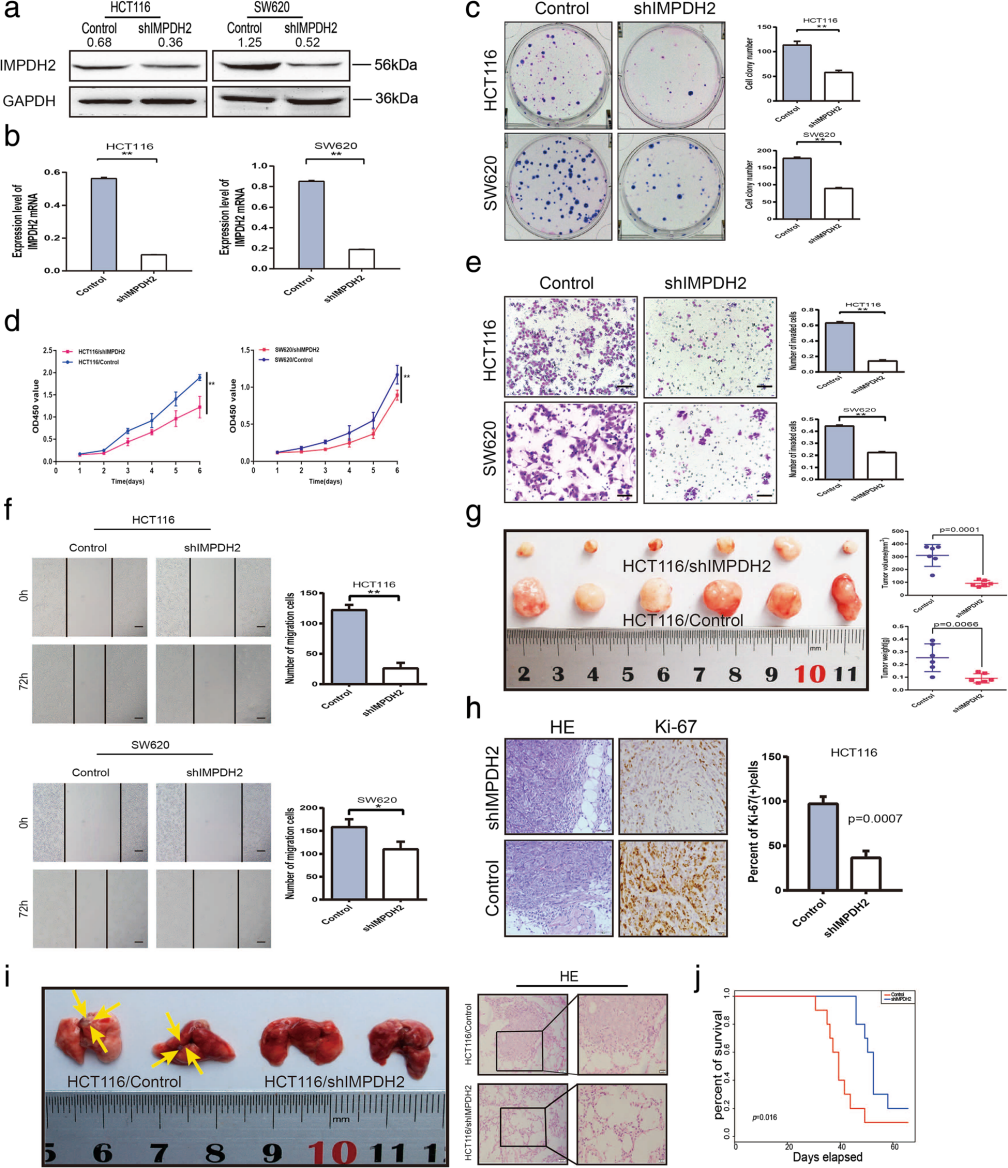
Downregulation of IMPDH2 inhibits proliferation, migration and invasion of CRC cells and suppresses tumour growth in the nude mouse model. (a and b) Knockdown of IMPDH2 was confirmed at the protein and mRNA level in HCT116 and SW620 cells by western blotting and qPCR. Mean ± SD (*n* = 3). **(c and d)** IMPDH2 knockdown inhibited proliferation ability of HCT116 and SW620 cells as determined by colony formation and CCK8 assays. Mean ± SD (*n* = 3). **(e)** IMPDH2 knockdown significantly suppressed the invasion ability of HCT116 and SW620 cells by the transwell assay. Representative photographs (left) and quantification (right) are shown. The number of cells that invaded through the extracellular matrix after 24 h was counted in five randomly selected microscopic fields. Mean ± SD (n = 3). Scale bars, 100 μm. **(f)** IMPDH2 knockdown significantly inhibited the migration ability of HCT116 and SW620 cells by cell wound healing assay. Images were taken at 0 h, 24 h, 48 h and 72 h. The number of migrated cells was counted (right). Mean ± SD (n = 3). Scale bars, 200 μm. **(g)** IMPDH2 silencing inhibited tumour growth in the nude mouse model by xenograft growth assay. Gross observation of xenograft tumour size (left). Statistical chart of a xenograft tumour volume and weight (right). **(h)** H&E and Ki-67 staining of a xenograft tumour. The percent of Ki-67 positive cells was shown (right). Scale bars, 50 μm and 20 μm. **(i)** Tumor cells were injected into nude mice through the tail vein to evaluate the lung homing potential of cells. Gross observation of lung metastases (left). H&E staining of lung metastatic nodules (right). Scale bars, 50 μm and 20 μm. **(j)** Kaplan-Meier survival analyses (log-rank) for the mice with HCT116/shIMPDH2 cells versus HCT116/Control cells were performed. Each error bar represents the mean ± SD of three replicate samples. *P < 0.05; **P < 0.01

To elucidate the impact of knockdown of IMPDH2 in CRC cells, endogenous IMPDH2 expression in HCT116 and SW620 cells was silenced using a lentiviral vector carrying a shRNA specifically targeting IMPDH2 (Fig. 3a and b). As shown in Figure3c and 3d, cell growth and proliferation abilities were significantly inhibited after downregulation of IMPDH2 (*p* < 0.05). Moreover, the invasive and migratory abilities were lower in IMPDH2-silenced cells than in the control cells (p < 0.05, Fig. 3e and f). Xenograft assays further uncovered that tumours formed by HCT116/shRNA cells grew much more slowly than tumours formed by HCT116/Scramble group (Fig. 3g). Palpable tumours were detected on the seventh day after injection (Fig. 3g). Tumours from the shRNA group were significantly smaller than those from the control group at day 28 (p < 0.05, Fig. 3g) . The result of IHC analysis confirmed that tumors generated from HCT16/shIMPDH2 cells exhibited lower Ki-67 index compared to tumors generated from HCT116/Scramble cells (Fig. 3h).

To further validate the effect of endogenous IMPDH2 on the potential of homing capacity in CRC, we respectively injected HCT116/Control cells and HCT116/shIMPDH2 cells into 10 pairs of nude mice through the tail vein to establish a mouse model for lung metastases of CRC. The nude mice were sacrificed after 8 weeks. We examined the number and size of tumor metastatic nodules under a microscope in the lung. As shown in Fig. 3i, compared with the control group in which four mice presented lung colonization, no lung colonization was observed in mice with HCT116/shIMPDH2 cells. In addition, it came out that knockdown of IMPDH2 led to longer overall survival of the animals (*P* < 0.05, Fig. 3j). The above findings indicate that IMPDH2 exerts its oncogenic roles by promoting the proliferation, invasion, migration and tumourigenesis of CRC cells.

### IMPDH2 promoted the invasion and metastasis of CRC cells through EMT

**Fig. 4:**
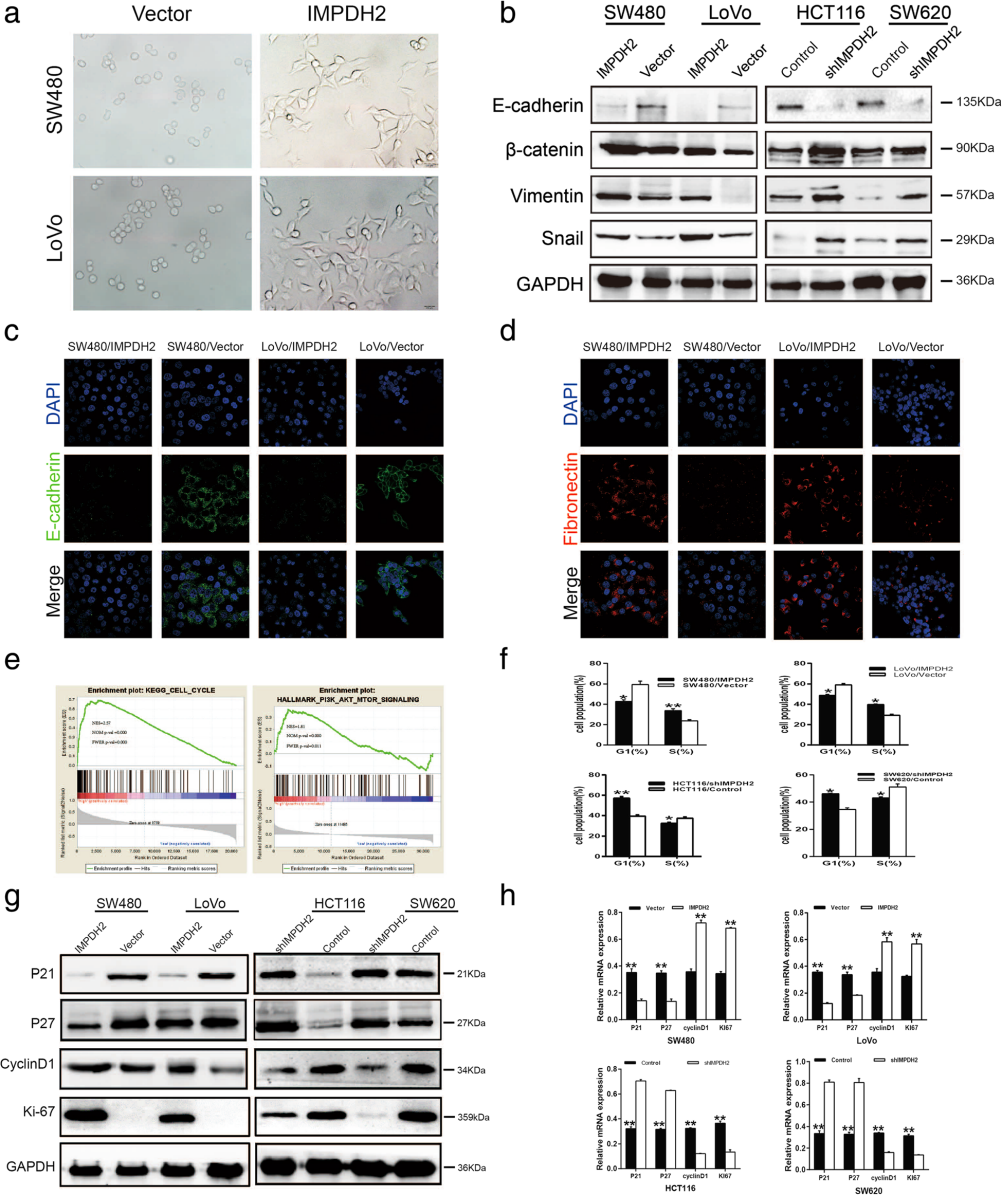
The effect of IMPDH2 on EMT and cell-cycle transition of CRC cells. (a) The spindle cell phenotype of IMPDH2-overexpressed cells (SW480/IMPDH2 and LoVo/IMPDH2) and the epithelial phenotype of CRC cells from its control group (Vector) showing epithelial-to-mesenchymal transition (EMT) induced by IMPDH2 overexpression. **(b)** IMPDH2 overexpression induces hallmarks of the EMT, including loss of E-cadherin and accumulation of Vimentin and Snail in CRC cells. **(c and d)** Immunofluorescent staining of E-cadherin and Fibronectin in SW480/IMPDH2 and LoVo/IMPDH2 Cells. **(e)** GSEA showing a significant association between IMPDH2 expression and CELL_CYCLE and PI3K_AKT_MTOR_SIGNALING signaling pathway. The top portion of the figure plots the enrichment scores for each gene, whereas the bottom portion of the plot shows the value of the ranking metric moving down the list of ranked genes. Y-axis: value of the ranking metric; X-axis: the rank for all genes. NES, normalized enrichment score. **(f)** Cells proportion in various phases of the cell cycle. Cells were stained with Propidium Iodide (PI) and analyzed by flow cytometry. Mean ± SD (n = 3). **(g)** Western blotting analysis of p21Cip1, p27Kip1, cyclin D1, and Ki-67 proteins in IMPDH2-overexpressed cells or IMPDH2 shRNA-infected cells. GAPDH was used as a loading control. *P < 0.05; **P < 0.01. **(h)** Real-time qPCR analysis of p21Cip1, p27Kip1, Ki-67 and cyclin D1 mRNA expression in IMPDH2-overexpressed cells (upper panel) or IMPDH2 shRNA-infected cells (lower panel). Expression levels were normalized to GAPDH. Mean ± SD (n = 3)

Based on the comparison between the IMPDH2-overexpressed cells (SW480/IMPDH2 and LoVo/IMPDH2) and their control groups, we found that IMPDH2 overexpression induced transdifferentiation of non-invasive epithelial cells to mesenchymal, spindle cells (Fig. 4a), demonstrating that IMPDH2 might be involved in epithelial-mesenchymal transition (EMT) of CRC cells, a crucial process characterized by tumor cell invasion and migration [19]. By means of stable transfection, we found that epithelial marker E-cadherin was downregulated, whereas mesenchymal markers Vimentin and Snail were upregulated in IMPDH2-overexpressed CRC cells (Fig. 4b). By contrast, E-cadherin was significantly increased in IMPDH2-silenced CRC cells, while Vimentin and Snail were decreased (Fig. 4b). The immunofluorescent assay further confirmed that E-cadherin was lowly expressed in the IMPDH2-overexpressed CRC cells while Fibronectin was highly expressed. (Fig. 4c and d). In addition, the invasive and migratory potential of SW480 and LoVo cells overexpressing IMPDH2 was increased, as detected by the transwell and wound-healing assay (Fig. 2e and f). However, the opposite phenomenon was observed on HCT116/shIMPDH2 and SW620/shIMPDH2 CRC cells (Fig. 3e and f). Moreover, lung metastases were found in the HCT116/Control cells (Fig. 3i). These results demonstrate that IMPDH2 could promote the invasion and metastasis of CRC cells via EMT.

### IMPDH2 accelerated the cell cycle transition in CRC cells

To explore the possible mechanism of IMPDH2 in CRC progression, gene set enrichment analysis (GSEA) was performed to compare the gene expression profiles of IMPDH2^low^ and IMPDH2^high^ CRC specimens. TCGA database containing 644 CRC tissues was divided into IMPDH2^low^ (*n* = 322) and IMPDH2^high^ (n = 322) groups based on the median expression level of IMPDH2. GSEA showed that the gene set of KEGG_CELL_CYCLE (cell cycle) (Fig. 4e) was significantly enriched in IMPDH2^high^ CRC specimens, indicating that this gene set could be significantly associated with elevated IMPDH2 expression in CRC.

To confirm the mechanism by which IMPDH2 promotes cell cycle transition of CRC cells, we performed flow cytometry analysis. The result uncovered that overexpressing IMPDH2 significantly increased the proportion of S phase cells and decreased the cells in the G0/G1 phase. In contrast, silencing IMPDH2 increased the percentage of G0/G1 phase cells and reduced S phase cells (Fig. 4f), implying that the G1/S phase transition might be accelerated by IMPDH2 in CRC cells.

On the ground that IMPDH2 expression appeared to be closely correlated with the G1/S phase transition of CRC cells, we further explored whether IMPDH2 could regulate cell cycle factors, including cyclin D1, p21Cip1 and p27Kip1. qPCR and western blotting analysis revealed a significant upregulation of cyclin D1 and Ki-67, in parallel with downregulation of p21Cip1 and p27Kip1 at the protein and mRNA level in IMPDH2-overexpressed cells compared to control cells (Fig. 4g and h). By contrast, a significant reduction of cyclin D1 and Ki-67 was observed in IMPDH2-silenced cells, whereas p21Cip1 and p27Kip1 were increased (Fig. 4g and h). Our results demonstrate that IMPDH2 accelerates the G1/S phase transition in CRC by modulating expression of cyclin D1, p21Cip1 and p27Kip1.

### IMPDH2 promoted CRC progression through the PI3K/AKT/mTOR and PI3K/AKT/FOXO1 signaling pathways

**Fig. 5:**
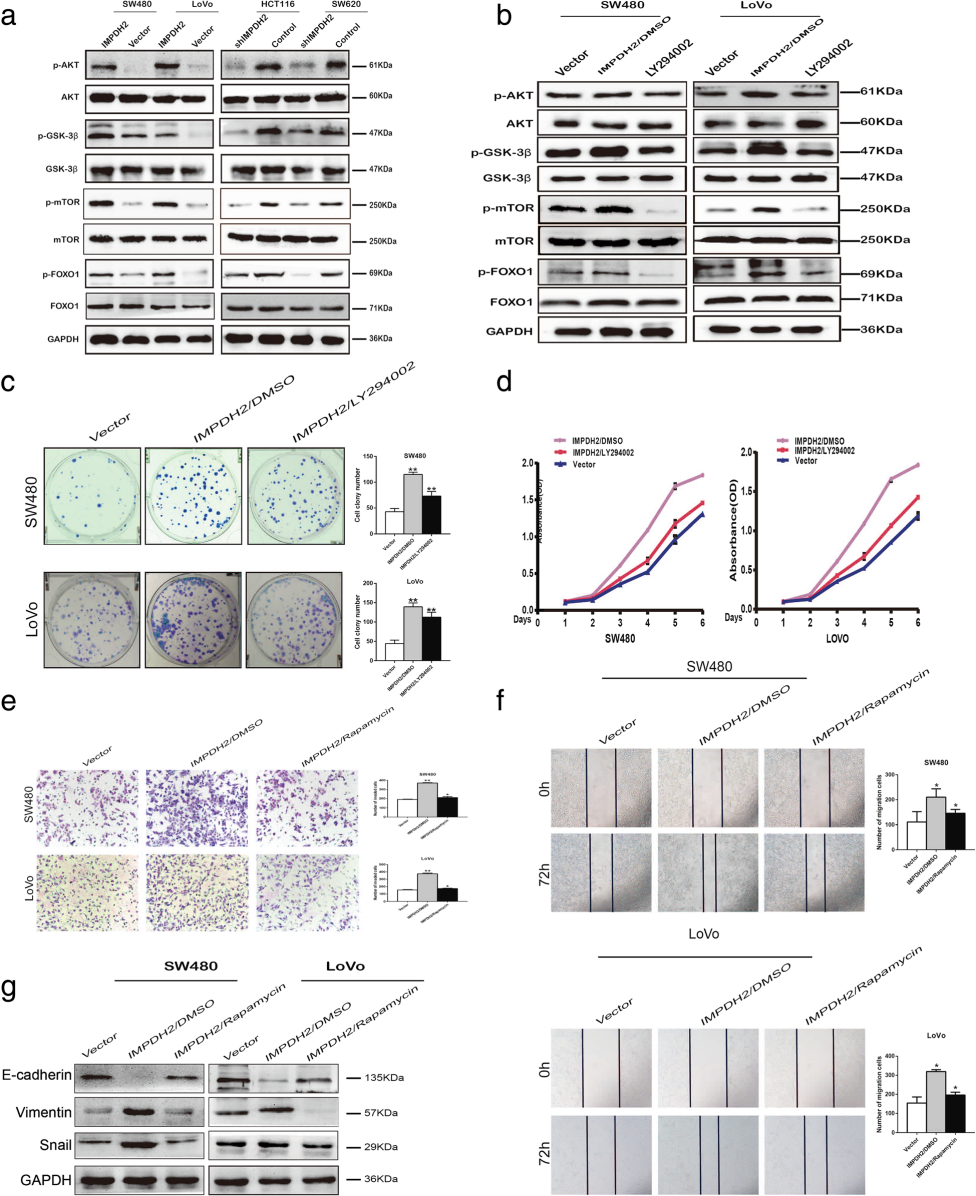
IMPDH2 promoted CRC progression through the PI3K/AKT/mTOR and PI3K/AKT/FOXO1 signaling pathways. (a) Western blotting analysis of p-AKT, total AKT, p-GSK-3β, total GSK-3β, p-mTOR, total mTOR, p-FOXO1, and total FOXO1 in IMPDH2-overexpressed cells or IMPDH2 shRNA-infected cells. **(b)** SW480/IMPDH2 and LoVo/IMPDH2 cells were treated with the AKT inhibitor LY294002 (20IM) and DMSO for 24 h, then harvested to examine the expression levels of the indicated proteins by Western blotting. **(c)** Colony formation assay after treatment with LY294002 and DMSO. Mean ± SD (n = 3). **(d)** The proliferation ability of SW480/IMPDH2 and LoVo/IMPDH2 cells were determined by CCK8 assay. *P < 0.05; **P < 0.01. **(e and f)** The invasive and migratory abilities of SW480/IMPDH2 and LoVo/IMPDH2 cells were determined by transwell and wound healing assays after treatment with rapamycin and DMSO. Mean ± SD (n = 3). *P < 0.05; **P < 0.01. **(g)** Inhibition of the mTOR activity induced upregulation of E-cadherin and downregulation of Vimentin and Snail in IMPDH2-overexpressed CRC cells

To gain further insight into the molecular mechanism by which IMPDH2 mediated oncogenic roles in CRC, we performed GSEA with TCGA data based on IMPDH2 expression. We found that one hallmark gene set, HALLMARK_PI3K_AKT_MTOR_SIGNALING (Fig. 4e), was enriched in IMPDH2^high^ CRC samples, demonstrating that this hallmark gene set were positively correlated with high expression of IMPDH2 in CRC. To our knowledge, mTOR and FOXO1 are key kinase downstream targets of PI3K/AKT signaling pathway, both of which are linked to cell cycle progression and cell proliferation [20, 21]. Therefore, we further investigated whether the proliferation-promoting effects of IMPDH2 were dependent on PI3K/AKT/mTOR and PI3K/AKT/FOXO1 signaling. As shown in Fig. 5a, phospho-AKT (p-AKT (Ser473)), phospho-GSK-3β (p-GSK-3β), phospho-mTOR (p-mTOR) and phospho-FOXO1 (p-FOXO1) proteins were increased by overexpressing IMPDH2 but decreased by its silencing. These results suggest that IMPDH2 might downregulate mTOR and FOXO1 transcriptional activity via activation of the PI3K/AKT signaling pathway. To address this hypothesis, we treated IMPDH2-overexpressed CRC cells with an AKT inhibitor (LY294002). We found that the expression levels of p-AKT, p-GSK-3β, p-mTOR and p-FOXO1 were significantly reduced by LY294002 in IMPDH2-overexpressed CRC cells (Fig. 5b). Additionally, we examined the proliferation ability of IMPDH2-overexpressed CRC cells by treatment with the AKT inhibitor. The colony formation and CCK8 assays showed that the proliferation of these IMPDH2-overexpressed cells was dramatically suppressed compared to control cells (Fig. 5c and d). These observations confirm that IMPDH2 could promote the proliferation of CRC cells by activation of the PI3K/AKT/mTOR and PI3K/AKT/FOXO1 pathways.

Previous articles have reported that the PI3K/AKT/mTOR signalling pathway played a crucial role in EMT process. Thus, to elucidate whether PI3K/AKT/mTOR pathway was involved in IMPDH2-induced invasion, migration and EMT in CRC cells, we treated the IMPDH2-overexpressed cells with the mTOR inhibitor rapamycin (10 μM). We found that inhibition of the mTOR activity induced suppression of cell invasion and migration, along with upregulation of E-cadherin and downregulation of Vimentin and Snail in IMPDH2-overexpressed cells (Fig. 5e, f and g).

## Discussion

In the current study, we highlighted that elevated expression of IMPDH2 was closely associated with several aggressive features and unfavorable prognosis of CRC patients. Overexpression of IMPDH2 could promote the proliferation, invasion, migration and tumorigenicity of CRC cells. Further studies uncovered that IMPDH2 exerted its oncogenic roles by promoting EMT and accelerating the G1/S phase transition in CRC. The above findings provide strong evidences to support the fact that IMPDH2 plays vital roles in the development and progression of CRC and may be a novel therapeutic target.

IMPDH is known as a key rate-limiting enzyme in de novo guanine nucleotide biosynthesis, the inhibitors of which is being widely used in cancer, immunosuppressive and anti-viral research and treatment [22–24]. Inhibition of IMPDH was capable of blocking cell-cycle progression in human T lymphocytes and suppressing the growth of human multiple myeloma cells [10, 25]. IMPDH2 is believed to be a fascinating target for cancer therapy due to its overexpression particularly in rapidly proliferating and neoplastic cells. A growing number of studies have demonstrated that IMPDH2 was closely implicated in cellular proliferation and tumorigenesis [4, 26–28]. Herein, we found that IMPDH2 was upregulated at the mRNA and protein level in CRC cell lines, in agreement with a previous study [17]. Then by data-mining in TCGA, we showed that IMPDH2 mRNA was significantly overexpressed in CRC tissues samples. Clinically, elevated expression of IMPDH2 in CRC tissues was further confirmed by qPCR, western blotting and immunohistochemistry analysis. Additionally, the statistical analysis revealed that high IMPDH2 expression significantly correlated with T stage, lymph node state, distant metastasis, lymphovascular invasion and clinical stage and was strongly associated with shorter survival of CRC patients. Moreover, multivariate analysis implied that lymph node state, distant metastasis and IMPDH2 expression could be independent prognostic factors for CRC patients. Our further in vivo and in vitro experiments revealed that IMPDH2 was critically involved in regulation of the proliferation, migration, invasion and tumorigenicity of CRC cells. In the light of these findings, we concluded that IMPDH2 may play oncogenic roles in CRC.

EMT is a key mechanism involved in the complex process of tumor invasion and metastasis. During cancer progression, EMT can alter adhesion of epithelial cancer cells and allow them to invade and migrate to the distant sites, thus contributing to tumor metastasis [29, 30]. Furthermore, EMT-linked loss of the basement membrane (BM) was closely linked to poor prognosis of CRC patients [30]. In the current study, we found that overexpressing IMPDH2 decreased the level of E-cadherin and increased that of Vimentin and Snail in CRC cells. On the contrary, silencing of IMPDH2 induced converse results. The results of immunofluorescent assay further revealed that E-cadherin was lowly expressed in the IMPDH2-overexpressed CRC cells while Fibronectin was highly expressed. These data demonstrate that IMPDH2 overexpression can promote the EMT of CRC cells.

Recently, microRNA-34a has been supposed to downregulate the GTP-dependent Ras signaling pathway by targeting IMPDH in different types of malignant cells [31]. Activation of the tumor suppressor p53 can inhibit cellular IMPDH2 activity and reduce cellular GTP level, thereby repressing cancer cell growth [32]. Furthermore, Inactivation of IMPDH triggers apoptosis to inhibit the growth of multiple myeloma cells primarily via a caspase-independent, Bax/AIF/Endo G pathway [16]. There is evidence that IMPDH2 interacts with the pleckstrin homology domain of PKB/AKT in the regulation of GTP biosynthesis [33]. Herein, we found that the gene set related to cell cycle positively correlated with elevated IMPDH2 expression by GSEA. It is well established that cell-cycle progression is one of the most predominant factors to promote cell proliferation. However, the underlying mechanisms of IMPDH2 involved in cell proliferation of CRC cells remain poorly elucidated.

Accumulating studies have revealed that the PI3K/AKT/mTOR pathway participates in regulating cellular events, such as cell growth, adhesion, migration and survival [34–37]. Activation of AKT signalling can contribute to cell proliferation and tumor progression by modulating its downstream cell cycle factors [38]. Furthermore, activated AKT induced the phosphorylation of various downstream targets, such as mTOR, FOXO1 and GSK-3β [39–41]. It has been validated that mTOR inhibitors induced cell cycle arrest and suppressed cell proliferation in EBV associated T- and NK-cell lymphomas [42]. Recent evidence has supported that inhibition of mTOR contributed to cell cycle arrest in prostate cancer radioresistant cells [39]. Intriguingly, based on GSEA by TCGA database, we found that HALLMARK_PI3K_AKT_MTOR_SIGNALING was significantly enriched in IMPDH2^high^ CRC specimens. By qPCR and western blotting, we observed that IMPDH2 could accelerate the G1/S phase transition of CRC cells by regulating expression of cyclin D1, p21Cip1 and p27Kip1. These findings drove us to hypothesize that IMPDH2 might promote cell cycle transition by targeting mTOR to regulate the expression levels of cell cycle regulators. It has been reported that AKT phosphorylation at both Ser473 and Thr308 residues, completely activates the AKT signaling pathway [43]. LY294002 is a small molecule that competitively and reversibly inhibits the ATP binding site of several different PI3Ks, and is a specific inhibitor of PI3K/AKT pathway. It results in suppression of tumor growth and induction of apoptosis in colon cancer cells, with decreased expression of phosphorylated AKT (Ser473) [44]. Thus, to further substantiate the above intriguing hypothesis, we examined the levels of p-AKT (Ser473) and p-mTOR. In our study, p-AKT and p-mTOR were found to be downregulated in IMPDH2-silenced CRC cells, but upregulated in IMPDH2-overexpressed CRC cells. Furthermore, increased expression of p-AKT and p-mTOR was significantly suppressed in IMPDH2-overexpressed CRC cells by treatment with AKT inhibitors, along with a significant decrease in cellular growth and colony formation.

Additionally, FOXO transcription factors were supposed to exert its oncogenic effect by regulating the expression of genes involved in diverse cellular processes including apoptosis, cell proliferation and genotoxic/oxidative stresses [45, 46]. Given that FOXO1 is one of cell cycle transition-related genes [21, 47, 48], we attempt to validate whether IMPDH2-mediated cell cycle transition is dependent on the PI3K/AKT/FOXO1 pathway. In the same manner, p-AKT and p-FOXO1 were detected to be markedly decreased in IMPDH2-silenced CRC cells, but increased in IMPDH2-overexpressed CRC cells. Furthermore, AKT inhibitors induced a significant decrease of p-AKT and p-FOXO1 in IMPDH2-overexpressed CRC cells, thereby resulting in cell growth arrest and inhibition of colony formation. These above observations suggest that IMPDH2-induced proliferation and tumorigenesis might be due to accelerating cell cycle transition via activation of the PI3K/AKT/mTOR and PI3K/AKT/FOXO1 pathways.

There is compelling evidence that EMT is mediated by regulating PI3K/AKT/mTOR pathway in some human tumors [49, 50]. Using the mTOR inhibitor rapamycin, we observed that inactivation of the mTOR pathway in IMPDH2-overexpressed CRC cells led to inhibition of cell invasion and migration. More importantly, the epithelial marker E-cadherin was significantly increased in IMPDH2-overexpressed CRC cells after inhibition of the mTOR activity, whereas the mesenchymal markers Vimentin and Snail were decreased. Therefore, our data support that activation of PI3K/AKT/mTOR signalling pathway was required for IMPDH2-induced invasion, migration and EMT of CRC cells.

## Conclusions

In summary, we have demonstrated that IMPDH2 is upregulated in CRC and closely associated with poor prognosis of CRC patients. Our study provides compelling evidence that IMPDH2 can promote the proliferation, invasion and migration, tumorigenicity and EMT of CRC cells. Furthermore, overexpressing IMPDH2 accelerated cell cycle transition by activating the PI3K/AKT/mTOR and PI3K/AKT/FOXO1 pathways and facilitate cell invasion, migration and EMT by regulating PI3K/AKT/mTOR pathway. These findings indicate that IMPDH2 has the potential as one of the most valuable prognostic and therapeutic biomarker for CRC.

## Abbreviations

CRC: Colorectal cancer
DAB: 3,3-diaminobenzidine
EMT: Epithelial-mesenchymal transition
FDR: False discovery rate
GSEA: Gene set enrichment analysis
H&E: Haematoxylin and eosin
IHC: Immunohistochemistry
IMP: Inosine monophosphate
IMPDH2: Inosine 5′-monophosphate dehydrogenase type II
NAD +: nicotinamide adenine dinucleotide.
qPCR: Quantitative real-time polymerase chain reaction
STR: Short tandem repeat
TCGA: The Cancer Genome Atlas
XMP: Xanthosine monophosphate
